# Supplementary material for: A Highly Sensitive and Specific SARS-CoV-2 Spike- and Nucleoprotein-Based Fluorescent Multiplex Immunoassay (FMIA) to Measure IgG, IgA, and IgM Class Antibodies
Source: Microbiol Spectr. 2021 Nov 17;9(3):e01131-21. doi: 10.1128/Spectrum.01131-21 (PMC8597651; doi:10.1128/Spectrum.01131-21)
Supplement: SUPPLEMENTAL FILE 1 — Supplemental material. Download SPECTRUM01131-21_Supp_1_seq8.pdf, PDF file, 0.3 MB [file spectrum01131-21_supp_1_seq8.pdf]

Supplemental data

A detailed description of the SARS-CoV-2 FMIA

Conjugation of microspheres. SARS-CoV-2 RBD (product code: REC31849, the Native Antigen Company), SFL (product code: REC31868, the Native Antigen Company) and nucleoprotein (product code: REC31812, the Native Antigen Company) antigens were conjugated on the surfaces of MagPlex®-C superparamagnetic carboxylated microspheres (Luminex) by carbodiimide reaction as described previously [1].

Preparation of plates. Serum samples, the WHO reference serum and controls were diluted to PBS solution (pH 7.2) containing 1% (v/v) Tween20, 1% (w/v) bovine serum albumin, 0.5% polyvinyl alcohol and 0.8% (w/v) polyvinylpyrrolidone. 25 µl aliquots of diluted samples, reference serum and controls were added on black 96-well plates (Costar 3915) with 25 µl of PBS containing the three microsphere regions (1750 microspheres/region/well) each conjugated with a single type of SARS-CoV-2 antigen. The plates were incubated in RT with shaking at 600 rpm in the dark for 1 hour, after which unbound particles were washed away with a magnetic plate washer (ELx405 and 405TSRS, BioTek) by 2 x 200 µl of PBS containing 0.05% (v/v) Tween20. 50 µl 1:100 diluted IgG, IgA or IgM detection antibody conjugate was added to the wells. Detection antibodies used were R-Phycoerythrin-conjugated Affinipure Goat Anti-Human IgG, IgA or IgM Fcγ Fragment Specific (product code for IgG: 109-115-098, product code for IgA: 109-115-011, product code for IgM: 109-116-129, Jackson Immuno Research). The plates were incubated for 30 minutes in RT, in the dark with shaking at 600 rpm, after which unbound particles were washed away as described above. 80 µl of PBS was added to the wells and the plates were incubated for 5 minutes in RT, in the dark with shaking at 600 rpm before measurement of antibody levels with MAGPIX® system (Luminex). IgG, IgA and IgM antibody levels were measured from separate plates.

Calculation of FMIA U/ml antibody concentrations. IgG, IgA and IgM antibody levels were determined as antibody concentrations (FMIA U/ml) interpolated from 5-parameter logistic (5-PL)

curves (xPONENT software version 4.2, Luminex) created from serially diluted (1:400–1:1638400) in-house reference sera each with two true duplicates. 1:400 dilution was given an arbitrary concentration of 100 FMIA U/ml. In addition to in-house reference serum and two blank wells, high and low concentration controls were included in each plate in true duplicates. The samples of the negative serum panel (n=402) were analyzed as true duplicates diluted 1:100. The samples of the positive serum panel (n=147) were analyzed as 1:100 and 1:1600 dilutions in true duplicates and the results were determined as the mean concentrations of two dilutions. Samples with antibody levels higher than the linear range of the reference curve were re-analyzed with a higher serum dilution. The antibody concentration values under the limit of detection were given a value of one-half of the limit of detection (LOD).

Evaluation of the analytical performance of FMIA.

Precision. The precision of FMIA was assessed by intra- and inter-assay variation as the percentage of the mean coefficient of variation (CV%) of antibody concentrations (FMIA U/ml). Intra-assay variation was determined as the mean CV% of all dilutions per antigen (n=70–91 replicates, 13 plates) and antibody class. Inter-assay variation was determined by comparing the levels of in-house reference serum analyzed on the same day (IgM n=5, IgA n=6, IgG n=8) in different plates (IgM n=10, IgA n=12, IgG n=17). In addition, inter-assay variation was assessed by analyzing the same five sera on five different days.

Intermediate precision. The reproducibility of the FMIA was assessed by comparing the results obtained by three laboratory technicians and as a variation caused by different batches of crucial reagents. All technicians analyzed the same 19 samples included in the negative and positive serum panels. Mean CV% was calculated for all technicians together. Crucial reagents assessed were conjugated microspheres and detection antibodies. For IgA detection antibodies, two batches were used in the analysis of 21 samples and the mean CV% was calculated. For the detection of IgG

antibodies, the sample sizes were 14 (N) and 33 (RBD and SFL). All IgM analyses were performed using the same batch of detection antibodies. The variation caused by different batches (n=4) of conjugated microspheres was assessed by a comparison of IgG antibody concentrations of two sets of microspheres at a time and then calculating the overall CV% per antigen between four batches. The number of samples differed between separate batch comparisons (n=13-31, 11-29 and 11-26 samples for N, RBD and SFL, respectively).

Calibration against WHO International Standard.

The IgG specific in-house reference serum was calibrated against WHO International Standard (NIBSC code 20/136 [2]). The IgG specific concentrations against antigens N, RBD and SFL of the in-house reference serum were determined in an assay with the WHO international standard analyzed in true duplicates with seven serial dilutions 1:100–1:1638400, with 1:100 being assigned with the concentration of 10 binding antibody units (BAU)/ml. The in-house reference serum was analyzed as 8 true duplicates each serially diluted 1:100–1:1638400, and the analysis was repeated on two days. The mean concentration for each dilution of the in-house reference serum (n=22, 30 and 40 for nucleoprotein, RBD and SFL, respectively) was interpolated from the linear range (1:1600-1:409600) of the WHO international standard. In addition, these values determined for the in-house reference serum were used in an assay to measure the antibody concentrations of 30 serum samples (true duplicates, dilutions 1:100 and 1:1600), which were compared to the antibody concentrations obtained using the WHO international standard. The mean CV% of antibody concentrations of the sera (n = 28, 25 and 51 for nucleoprotein, RBD and SFL, respectively) was interpolated from the linear range of the WHO international standard, and the mean CV% of antibody concentrations below 20% and correlation $R^2 \geq 0{,}95$ was considered acceptable. Calibration factor was obtained for each antigen separately and used to convert FMIA U/ml results into BAU/ml.

Supplemental figures and tables

Supplementary Figure S1. Distribution of positive serum panel’s days post-onset of symptoms at the time of sample collection.

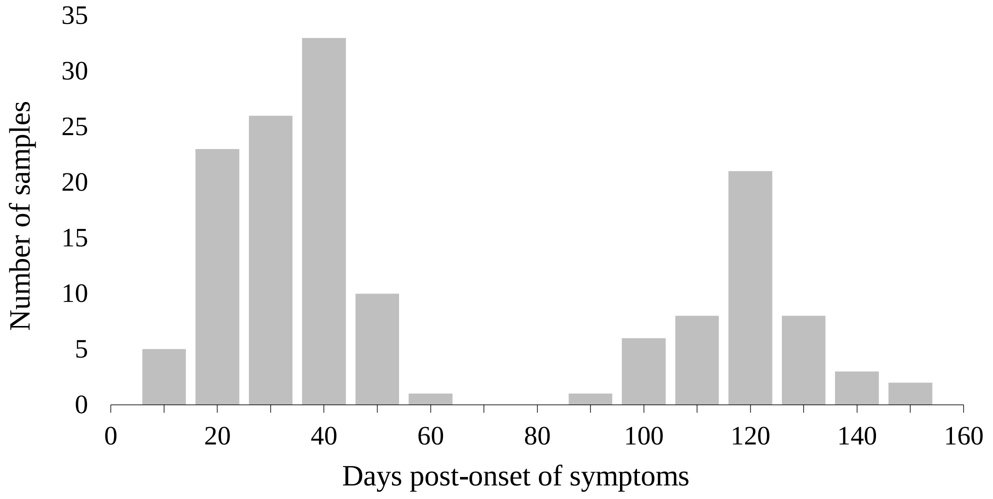

Supplementary Figure S2. Spearman correlation (ρ) and significance (p) between IgA and IgM specific FMIA U/ml and EIA unit results. Dashed lines mark thresholds for positivity per antigen. S1 = SARS-CoV-2 spike glycoprotein S1 subunit, RBD = receptor binding domain of SARS-CoV-2 (Wuhan-Hu-1) spike glycoprotein. SFL = full-length spike glycoprotein of SARS-CoV-2 (Wuhan-Hu-1). One point may represent multiple samples (n=80).

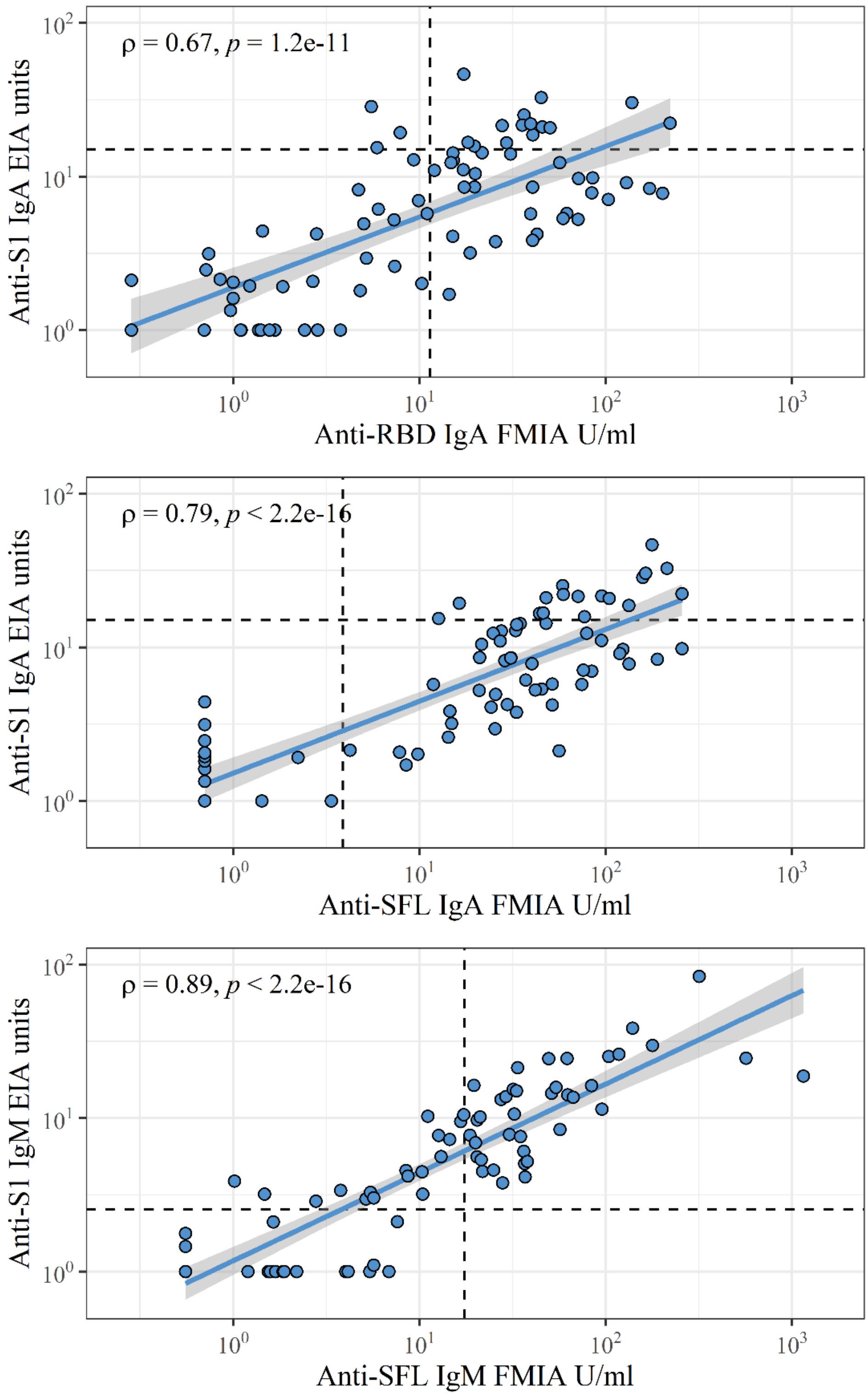

Supplementary Table S1. Age and sex distributions of the participants that donated sera to pre-COVID-19 pandemic (negative serum panel, n=402), as part of the COVID-19 household study (positive serum panel, n=58) and as part of the Jalkanen et al. (2021) study [3] used in FMIA and EIA comparisons.

	Validation of CoV-2 FMIA				FMIA and EIA comparisons			
	Negative serum panel		Positive serum panel		Vaccinated HCWs		Patients	
Age group (years)	n (%)	% female	n (%)	% female	n (%)	% female	n (%)	% female
1-10	100 (25)	50	3 (5)	0	-	-	-	-
11-20	38 (9)	50	7 (12)	57	-	-	-	-
21-30	36 (9)	53	5 (9)	20	2 (10)	100	5 (25)	40
31-40	38 (9)	50	13 (22)	62	6 (30)	100	3 (15)	67
41-50	38 (9)	50	20 (34)	65	9 (45)	89	2 (10)	50
51-60	38 (9)	50	8 (14)	63	2 (10)	50	4 (20)	25
61-70	38 (9)	50	2 (3)	50	1 (5)	100	6 (30)	50
71-80	38 (9)	50	-	-	-	-	-	-
81-90	38 (9)	50	-	-	-	-	-	-

HCWs = Healthcare workers

Supplementary Table S2. Limits of quantification and detection of FMIA.

Class	Antigen	LOQ	LOD
IgG	N	0.0048*	0.0094*
	RBD	0.0023*	0.0057*
	SFL	0.0046*	0.012*
IgA	N	0.03**	0.05**
	RBD	0.25**	0.57**
	SFL	0.59**	1.41**
IgM	N	0.11**	0.27**
	RBD	0.15**	0.36**
	SFL	0.48**	1.11**

LOQ = limit of quantification, LOD = limit of detection,* = WHO-standard adjusted LOQ and LOD in BAU/ml, ** = non-adjusted LOD and LOQ in FMIA U/ml. N = SARS-CoV-2 (Wuhan-Hu-1) nucleoprotein, RBD = receptor binding domain of SARS-CoV-2 (Wuhan-Hu-1) spike glycoprotein, SFL = full length spike glycoprotein of SARS-CoV-2 (Wuhan-Hu-1).

Supplementary Table S3. Intra- and inter-assay variation of FMIA for each antibody class and antigen.

Antibody class	Antigen	Intra-assay variation (CV%)[a]	Inter-assay variation (CV%)	
			Within a day[b]	Between days[c]
IgG	N	7	6	15
	RBD	8	4	11
	SFL	8	2	11
	Mean	8	4	12
IgA	N	7	2	6
	RBD	13	9	13
	SFL	13	20	7
	Mean	11	10	9
IgM	N	10	3	12
	RBD	10	4	7
	SFL	9	5	7
	Mean	10	4	9

CV = coefficient of variation, N = SARS-CoV-2 (Wuhan-Hu-1) nucleoprotein, RBD = receptor binding domain of SARS-CoV-2 (Wuhan-Hu-1) spike glycoprotein, SFL = full length spike glycoprotein of SARS-CoV-2 (Wuhan-Hu-1), [a] = Calculated from antibody concentrations of in-house standards that were analysed in 13 assays as seven serial dilutions (1:400–1:1638400) each with two replicates. Results are the mean CV% of replicates within plates. [b] = In-house standard’s variation within one day. The mean of two replicates was used in the calculation of CV% between plates. Depending on the antibody class, values are based on 5–8 days and 10–17 assays (IgM: 5 days and 10 assays, IgA: 6 days and 12 assays, IgG: 8 days and 17 assays). c = Five sera analysed on five days as two replicates. The mean of two replicates was used in calculations of CV% between days.

Supplementary Table S4. Positive serum panel and the number and proportion of samples positive in MNT and FMIA.

	Number of samples		Percentage of FMIA positive samples also positive in MNT (number of all FMIA positive samples)		
DPO	All	MNT positive	IgG positive*	IgA positive	IgM positive
<13	7	2	100% (3)	100% (3)	100% (3)
13-20	21	20	100% (21)	100% (21)	100% (21)
21-28	18	18	100% (18)	100% (18)	100% (18)
29-36	30	30	100% (30)	100% (30)	90% (27)
37-51	22	22	100% (22)	95% (21)	73% (16)
52-150	49	45	100% (49)	49% (24)	38% (21)
Total	147	137	100% (143)	82% (117)	73% (106)

FMIA = fluorescent multiplex immunoassay, MNT = microneutralization test, DPO = days post-onset of symptoms at sample collection. * = Determined with spike glycoprotein antibody thresholds.

Supplemental references

1. Ekström N, Virta C, Haveri A, et al. Analytical and clinical evaluation of antibody tests for SARS-CoV-2 serosurveillance studies used in Finland in 2020. medRxiv. 2021; 2021.01.21.21250207.
2. Mattiuzzo G, Bentley EM, Hassall M, et al. Establishment of the WHO International Standard and Reference Panel for anti-SARS-CoV-2 antibody. WHO/BS/20202402.
3. Jalkanen P, Kolehmainen P, Häkkinen HK, et al. COVID-19 mRNA vaccine induced antibody responses against three SARS-CoV-2 variants. Nat Commun. 2021; 12(1):3991.
